# Utility of the Hebb–Williams Maze Paradigm for Translational Research in Fragile X Syndrome: A Direct Comparison of Mice and Humans

**DOI:** 10.3389/fnmol.2018.00099

**Published:** 2018-03-28

**Authors:** Isabelle Boutet, Charles A. Collin, Lindsey S. MacLeod, Claude Messier, Matthew R. Holahan, Elizabeth Berry-Kravis, Reno M. Gandhi, Cary S. Kogan

**Affiliations:** ^1^School of Psychology, University of Ottawa, Ottawa, ON, Canada; ^2^Department of Neuroscience, Carleton University, Ottawa, ON, Canada; ^3^Pediatrics, Biochemistry, and Neurology, Rush University Medical Center, Chicago, IL, United States

**Keywords:** Fragile X syndrome, human, mouse, spatial learning, Hebb–Williams maze, translational research

## Abstract

To generate meaningful information, translational research must employ paradigms that allow extrapolation from animal models to humans. However, few studies have evaluated translational paradigms on the basis of defined validation criteria. We outline three criteria for validating translational paradigms. We then evaluate the Hebb–Williams maze paradigm (Hebb and Williams, 1946; Rabinovitch and Rosvold, 1951) on the basis of these criteria using Fragile X syndrome (FXS) as model disease. We focused on this paradigm because it allows direct comparison of humans and animals on tasks that are behaviorally equivalent (criterion #1) and because it measures spatial information processing, a cognitive domain for which FXS individuals and mice show impairments as compared to controls (criterion #2). We directly compared the performance of affected humans and mice across different experimental conditions and measures of behavior to identify which conditions produce comparable patterns of results in both species. Species differences were negligible for Mazes 2, 4, and 5 irrespective of the presence of visual cues, suggesting that these mazes could be used to measure spatial learning in both species. With regards to performance on the first trial, which reflects visuo-spatial problem solving, Mazes 5 and 9 without visual cues produced the most consistent results. We conclude that the Hebb–Williams mazes paradigm has the potential to be utilized in translational research to measure comparable cognitive functions in FXS humans and animals (criterion #3).

## Introduction

“*By carefully selecting tasks for animals with high construct validity to human tasks, reliability and accuracy of translational efforts will not be lost and meaningful progress can be made in ameliorating the cognitive deficits that affect the lives of those suffering from mental illness*.”

(Gilmour et al., 2013, p. 2126).

The quotation above articulates the potential benefits of translational research for society and stresses the need to carefully select tasks that allow extrapolation from animal models to humans in order to reap these benefits. Surprisingly, there is little consensus on what criteria should be used for validating translational paradigms, a fact that significantly hinders selection of appropriate tasks (see Willner, 1986 for validation criteria for animal models) and therefore limits the likelihood that laboratory research will translate to human treatments. We address this limitation by outlining three criteria for validating translational paradigms. We then use these criteria to evaluate the Hebb–Williams maze paradigm using Fragile X syndrome (FXS) as a model disease.

Fragile X syndrome is the most common identifiable genetic cause of intellectual disability and a leading genetic cause of autism spectrum disorder (ASD) (e.g., Hagerman, 1987; Farzin et al., 2006). The disorder arises from mutation of a single gene, the FMR1 (Fragile X Mental Retardation 1) gene, which codes for a protein that plays a key role in experience-dependent synaptic plasticity (e.g., Huber et al., 2002). The mutation is caused by intergenerational expansion of a trinucleotide region upstream of the coding sequence results in methylation and silencing of the FMR1 gene [Online Mendelian Inheritance in Man^®^ (OMIM) 309550^1^; Verkerk et al., 1991). The silencing of the FMR1 gene results in several significant behavioral and cognitive impairments including deficits in attention (Backes et al., 2002; Baumgardner et al., 1995), visual-spatial cognition (Crowe and Hay, 1990; Cornish et al., 1998, 1999), working memory (Schapiro et al., 1995; Jakala et al., 1997), and visual-perceptual processing (Kogan et al., 2004a). Because it is the outcome of a single gene defect, FXS offers a remarkable opportunity to investigate the validity and feasibility of translational paradigms by comparing the animal model with affected individuals. The animal model for the disease, the *fmr1* knock-out (KO) mouse, has significantly contributed to our understanding of the neurobiology and synaptic mechanisms of the disorder and to the identification of potential therapeutic agents (e.g., Berry-Kravis et al., 2011). Despite these advances, translation of drug discovery research from the mouse model to humans has proven difficult (e.g., Arbaclofen trial: Berry-Kravis et al., 2017), highlighting the need for the identification of valid translational tasks.

Drawing from the literature (Willner, 1986; Gilmour et al., 2013; McGonigle and Ruggeri, 2014; Gabel et al., 2016), we propose that to allow for appropriate extrapolation from animal to human studies, translational paradigms must: (1) allow direct comparison of humans and animals on tasks that are behaviorally equivalent, (2) measure constructs that are fundamental to the disorder, and (3) engage comparable underlying neural mechanisms and cognitive functions in both species^2^. To our knowledge, only three studies have directly compared the performance of FXS humans and KO mouse on behaviorally equivalent paradigms (Frankland et al., 2004; Koekkoek et al., 2005; MacLeod et al., 2010; see, e.g., Gilmour et al., 2013; Gabel et al., 2016 for other conditions). Two studies (Frankland et al., 2004; Koekkoek et al., 2005) employed paradigms that measure prepulse inhibition, a function that is thought to be impaired in FXS as indicated by heightened sensitivity to sensory stimulation (mouse: Chen and Toth, 2001; humans: Miller et al., 1999). The other study employed a paradigm that measures spatial processing (MacLeod et al., 2010), a central feature of cognitive impairment in affected individuals (e.g., Crowe and Hay, 1990; Cornish et al., 1998, 1999; Kogan et al., 2004a) and the murine model (e.g., Baker et al., 2010; Gandhi et al., 2014b, but see Fisch et al., 1999). We review their findings below.

Frankland et al. (2004) measured prepulse inhibition to acoustic startle in *fmr1* KO mice, affected individuals and their respective comparison groups. Whereas prepulse inhibition was enhanced in KO mice as compared to controls, it was significantly reduced in humans. The authors concluded that the *FMR1/fmr1* gene impacts sensory processing in both species. However, because the results were opposite in direction for mice as compared to humans, it is unclear whether different mechanisms might be measured by this paradigm across the two species. Koekkoek et al. (2005) used an eye blink reflex paradigm to measure prepulse inhibition. They report a comparable reduction in prepulse inhibition in FXS humans and KO mice as compared to their respective comparison groups. This eye blink reflex paradigm has also been used in translational research to successfully demonstrate efficacy of a metabotropic glutamate receptor antagonist (i.e., Fenobam) for reversing prepulse inhibition in affected humans (Berry-Kravis et al., 2009). While these results point to the utility of the eye blink paradigm for translational research, it measures a discrete sensorimotor gating function that provides limited insight in to the central cognitive impairments in FXS.

MacLeod et al. (2010) used the Hebb–Williams mazes paradigm to measure visuo-spatial abilities in *fmr1* KO mice, affected individuals, and their respective comparison groups. Hebb–Williams mazes require successful navigation through different mazes whose configurations can be varied to provide problems of varying difficulty (Rabinovitch and Rosvold, 1951; Meunier et al., 1986). MacLeod et al. (2010) employed this paradigm because both traditional animal-based and computer versions of the task exist, allowing researchers to test mice and humans under equivalent conditions (Shore et al., 2001). Moreover, performance on the mazes appears to be dependent on the integrity of brain areas with known impairment in FXS humans and KO mice. Lesion studies in mice have demonstrated that successful performance on paradigms such as the radial arm maze, T-maze, and water maze rely on intact hippocampal processing (Mitchell et al., 1982; Morris et al., 1982; Hock and Bunsey, 1998; Okada and Okaichi, 2009). Equivalent results are found with similar visuo-spatial tasks in humans (Rogers and Kesner, 2006; Hunsaker et al., 2008). Structural abnormalities of the hippocampus are seen in FXS patients (see Bostrom et al., 2016 for a review). Although similar gross morphological differences have not been observed in the Fmr1 KO mice, significant neuronal pathology has been described in the form of longer dendritic spines in pyramidal cells in subfield CA1 (Grossman et al., 2006), smaller intra-infra pyramidal mossy fiber terminal fields (Mineur et al., 2002), as well as shorter dendrites, fewer dendritic spines and functional synaptic connections (Braun and Segal, 2000). Studies of human hippocampal neuronal dysmorphology reveal a higher density of dendritic spines suggestive of abnormal synaptic pruning in FXS (Irwin et al., 2000). Another area involved in maze performance is the posterior parietal cortex. This area is recruited during spatial navigational tasks in humans (Hyvarinen and Poranen, 1974; Andersen and Buneo, 2002; Spiers and Maguire, 2007) and mice (Harvey et al., 2012). Kogan et al. (2004b) demonstrated that visual-spatial processing associated with the posterior parietal cortex is selectively impaired in humans with FXS. Finally, the paradigm assesses cognitive functions that are related to the clinical features of the disorder and that are the principle targets for interventions. MacLeod et al. (2010) found comparable impairments on the mazes for both KO mice and affected humans which manifested as a lack of improvement in performance across trials, indicative of a spatial learning deficit. At the molecular level, Gandhi et al. (2014a) showed that a metabotropic glutamate receptor antagonist (i.e., MPEP) reversed these deficits in KO mice.

These findings suggest that the Hebb–Williams paradigm meets two of the criteria for validating translational paradigms: it allows for direct comparison of humans and animals on a paradigm that is behaviorally equivalent (criterion #1), and it measures cognitive functions and underlying neural mechanisms that are fundamental to the disorder (criterion #2). In addition, unlike other cognitive tasks, the Hebb–Williams maze paradigm is not too difficult for individuals with FXS to complete nor does it produce unacceptable levels of variability (Berry-Kravis et al., 2006). In the present study, we evaluated whether the paradigm meets criterion #3, with an emphasis on whether the paradigm engage comparable underlying cognitive functions in both species. We directly compared the performance of FXS humans and KO mice across different experimental conditions and measures of behavior. Our goal was to identify which conditions, if any, yield similar performance and hence which conditions might be used to measure comparable underlying cognitive functions in both species. This *direct comparison* approach complements the *homology of impairments* approach (Sjoberg, 2017) adopted by the aforementioned studies where mutant mice were compared to wild-type controls and FXS individuals were compared to typically developing participants or those matched for intellectual ability. While the homology of impairments approach provides valuable information for the identification of key features of the disorder, it is less appropriate for identifying tasks that engage comparable underlying mechanisms across species. Indeed, this approach assumes that reduced performance of affected participants compared to controls on equivalent tasks reflects impairments in the same underlying mechanism(s) in humans and animals (Sjoberg, 2017). This assumption might not always be valid for the following reasons. First, there are difficulties inherent to matching comparison groups to participants affected by FXS. Human studies typically match on the basis of chronological age or intellectual ability (e.g., Frankland et al., 2004; Kogan et al., 2004a, 2009; Lightbody et al., 2006; MacLeod et al., 2010; Klusek et al., 2015; McDuffie et al., 2015). Such techniques limit test implementation and interpretation of group effects. For example, choosing chronological age matched comparisons might yield ceiling effects for the unaffected group or floor effects in the FXS group. Moreover, impairments in the FXS group might be better explained by differences in understanding instructions rather than an impairment in the cognitive function of interest. On the other hand, similarities and/or differences between FXS individuals and mental-age matched comparisons, who are typically younger, might be better explained by differences in their stage of development rather than serve as evidence for preservation or impairment in a specific cognitive domain. Similarly, in mouse studies, there is no universal methodology for matching the comparison group to the KO group on the basis of intellectual abilities. Instead, behavior of KO mice is typically compared to that of age-matched wild type littermates, making it impossible to determine whether group differences arise from general cognitive impairments or from impairment of a distinct cognitive function. Second, the neuroconstructivist view of development (reviewed by Karmiloff-Smith, 1998, 2009) suggests that when there are no differences in performance in special populations vs. typically developing individuals, one cannot necessarily conclude that cognitive function is normal in the special population because the two groups may rely on different underlying mechanisms to perform the task. Differences in underlying mechanisms may arise because alterations at the level of gene expression (e.g., see Kooy, 2003; Errijgers and Kooy, 2004) could compensate for perturbations in the course of development such that affected individuals demonstrate normal or near normal performance at the behavioral level. It is also possible that affected individuals compensate by employing different neuronal structures to achieve the same outcome as typically developing individuals. Such considerations limit inferences that can be drawn on the basis of the homology of impairments approach, especially with respect to the measure of equivalent underlying mechanisms in humans and animals.

In the present study, we adopted a direct comparison approach to address these limitations. Indeed, to be informative and ultimately predictive of therapeutic response, translational paradigms should measure the same underlying construct across species (Willner, 1986; Gilmour et al., 2013). While there is no perfect solution, introducing a procedural modification or new variable to a paradigm with the goal of examining whether both species react similarly to this modification provides compelling evidence that equivalent underlying mechanisms are being measured (Willner, 1986; Shore et al., 2001; van der Staay et al., 2010; Sjoberg, 2017). This approach circumvents some of the limitations associated with the homology of impairments approach. It is important to note that we conceptualize these two approaches as complementary in yielding evidence in favor of the validity of a specific translational paradigm. We do not question the valuable contribution made by studies adopting the homology of impairments approach but instead highlight limitations of this approach to advancing the field of translational research. An optimal outcome is one whereby a behaviorally equivalent paradigm is first shown to measure key features of the disease by yielding homologous performance differences between comparison and affected participants across species, followed by a direct comparison of humans and animals on variations of the paradigm to identify conditions yielding comparable patterns of performance, which would suggest that the paradigm taps into comparable underlying mechanisms.

Having already established the potential for the Hebb–Williams mazes to serve as a translational paradigm using a homology of impairments approach (MacLeod et al., 2010), we sought complementary evidence in the present study using a direct comparison approach. We compared the profile of performance of FXS participants with that of KO mice on two variants of the Hebb–Williams paradigm and on multiple measures of performance. We compared data obtained on the standard version of the paradigm (MacLeod et al., 2010) as well as new data obtained on a variation with visual cues. We also performed multiple new analyses to directly compare humans and mice on various indicators of cognitive function and performance. Performing multiple cross-species comparisons across experimental conditions and measures (Vorhees and Williams, 2014) allowed us to identify which conditions, if any, produce comparable patterns of performance across species. As such, the present study seeks to identify which conditions of the paradigm meet *all* of the validation criteria outlined herein by accruing new data with respect to criterion three. This study therefore seeks to validate the Hebb–Williams maze paradigm for use in translational and drug discovery research and to provide practical information regarding which conditions and measures offer the best potential for extrapolating from mice to humans.

Notwithstanding the variable nomenclature and experimental manipulations that have been used to describe and investigate the impact of visual cues on spatial learning, evidence from both animals and humans suggest that the presence of visual cues in maze environments improves learning in typically developing participants (Heft, 1979; Jansen-Osmann and Fuchs, 2006; Waller and Lippa, 2007; reviewed by Chan et al., 2012). However, in certain populations visual cues may be ignored rather than being used to assist navigation (Wilkniss et al., 1997; Moffat and Resnick, 2002; Hanlon et al., 2006; Robaey et al., 2016). Particularly relevant to FXS, Robaey et al. (2016) showed that children who exhibit symptoms of Attention Deficit Hyperactivity Disorder (ADHD) do not rely on visual cues while navigating in an eight-arm radial maze. Because a majority of males with Fragile X exhibit symptoms of ADHD (e.g., Hagerman, 1987; Hatton et al., 2002; Farzin et al., 2006; Sullivan et al., 2006), introducing visual cues to the Hebb–Williams paradigm provides a critical experimental variation upon which to compare humans and mice. We also conducted new analyses to determine whether the two species display comparable patterns of results for the two main processes involved in solving mazes, namely basic visual functioning necessary for solving a novel spatial task and learning/memory. Indeed, while performance on the first trial is thought to reflect visuo-spatial processing and problem solving (Hebb and Williams, 1946), performance on subsequent trials reflects memory for the configuration of the maze and goal location as indicated by rate of learning across trials. Finally, we examined the relative difficulty of the mazes across the two species (Meunier et al., 1986). Level of difficulty is an index of problem-solving complexity and comparable patterns would suggest similar approaches to maze problem-solving in both species.

## Materials and Methods

### Mice

Two groups of male FVB.129P2-*Fmr1^tm1Cgr^*/J mice (JAX Stock #004624) mice were obtained from Jackson Laboratories (Bar Harbor, Maine, United States). The first group of mice provided data which has not been previously published on the paradigm with visual cues. The second group corresponds to mice tested in MacLeod et al. (2010) on the standard paradigm. Each strain had been backcrossed for 11 generations. Mice are pigmented (gray), do not carry the *rd1* mutation and possess the wild type *Pde6b* allele indicating that they do not suffer from blindness due to retinal degeneration. For the standard group, 11 animals were shipped at 4 weeks of age and were tested when they were approximately 3 months old. For the visual cue group, which was tested a year after the standard group, 10 animals were tested when they were approximately 5 months old. Eight days prior to behavioral testing, all subjects were individually housed in a climate-controlled vivarium (20–22°C) that was maintained on a 12 h light-dark cycle with lights on from 0700 to 1900. All testing was conducted during the light phase of the cycle. Mice were fed Harlan Global Rodent Chow and tap water. They were maintained at 85–90% of their *ad lib* body weight. Mice were weighed daily and fed their individually weighed ration of food 30 min after completion of the session. The mice were treated in accordance with the guidelines and principles set by the Canadian Council on Animal Care and tested under a protocol approved by the University of Ottawa Animal Care Committee.

### Mouse Apparatus

Mice were tested using the Hebb–Williams maze apparatus as described by Meunier et al. (1986). The apparatus was constructed using black opaque Plexiglas and was covered with a clear Plexiglas top (Plastics of Ottawa Ltd., Ottawa, ON, Canada). It consisted of a square open field (60 cm × 60 cm × 10 cm) with start and goal box compartments (20 cm × 10 cm × 10 cm) located at diagonally opposite corners. These compartments were fitted with clear Plexiglas lids that were attached with hinges and could be blocked with removable clear Plexiglas barriers. The goal box was fitted with a ledge (8 cm × 2.5 cm) with a recessed food cup in the center (2.5 cm diameter). The floor of the maze was divided into 36-equal squares that were clearly outlined in white. The squares were used as markers for placing the barriers in different maze configurations and to define error zones. The same maze configurations as in MacLeod et al. (2010) were employed, namely mazes 2, 4, 5, 8, 9, 11, and 12 (Rabinovitch and Rosvold, 1951). Testing was in the following order: Maze 12, 2, 8, 4, 5, 9, and 11. Pilot data with humans from our laboratory suggested that the other mazes were too easy and might yield ceiling effects. The order was determined on the basis of maze difficulty (less to more difficult) as assessed using pilot data. Removable barriers (10 cm high) were created using black opaque Plexiglas and each was supported by two permanent bases (2.5 cm × 2.5 cm). Extra-maze cues were minimized by conducting the study in an all-black enclosure and by having a dim light as the only source of illumination.

For the visual cue group, six images of simple geometric shapes (e.g., circle, square, and triangle) surrounded by a white background were added to each of the mazes (10 cm × 10 cm). An identical set of six shapes was used in each maze configuration. Each set of shapes was the same color as that of the test maze with the white background clearly distinguishing the shape from the maze wall. The visual cues were distributed within a test maze such that at least one image was visible from any position within the maze. Visual cues were laminated and adhered to the interior of the maze with double-sided tape.

### Mouse Procedure

**FIGURE 1:**
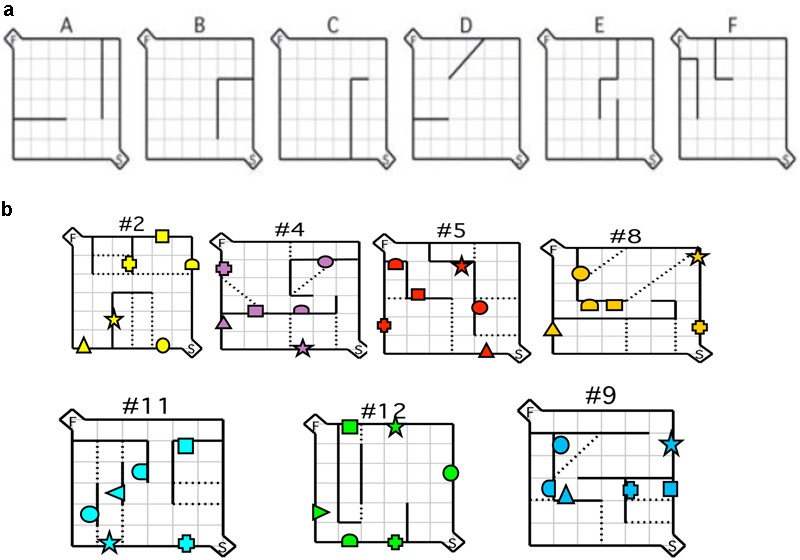
Maze configurations. **(a)** Testing was conducted using the six practice mazes **(A–F)** and **(b)** the seven test mazes depicted. For each maze configuration, the (S) depicted in the bottom right hand corner represents the start box, and the (F) in the top left corner represents the goal box. Error zones are depicted by the dotted lines. Location of visual cues is depicted by geometric shapes. Reprinted in part from MacLeod et al. (2010). A comparative study of the performance of individuals with Fragile X syndrome and Fmr1 knockout mice on Hebb–Williams mazes. *Genes, Brain and Behavior, 9*(1), 53–64.

The protocol consisted of three consecutive phases: habituation, acquisition, and testing. Initially, mice were habituated to the maze environment for 20 min per day on 4 consecutive days with barriers and doors to the start and goal box removed. During the last two sessions the goal box was baited (Rodent Chow, 100 mg) and mice had *ad lib* access to the food for the duration of the session. Subsequently, mice from both groups were trained on six acquisition mazes without visual cues as described by Rabinovitch and Rosvold (1951) (**Figure 1a**). On any given day, mice were tested such that they completed five trials for each of two of the six acquisition mazes. Mice completed all six acquisition mazes in sequence as many times as necessary for them to attain the criterion performance, which was defined as two consecutive sessions completed successfully in less than 30 s each. The acquisition phase required an average of 7 days to complete. On each acquisition trial, mice received a small reinforcer (Rodent Chow, 20 mg). Immediately following acquisition, mice were given a selection of the standard test mazes over 4 days (Rabinovitch and Rosvold, 1951) according to the same training protocol used during acquisition sessions. None of the acquisition or testing sessions exceeded 180 s. Mice completed the Rabinovitch and Rosvold (1951) maze configurations. Latency and number of errors were recorded. Latency was recorded from the moment the barrier at the start box was removed until the animal took its first bite of food. An error was scored each time the animal’s two front paws crossed into an error zone (**Figure 1b**). Experimenters were blind to the animal genotypes and were never visible to the mice during the runs. The maze was thoroughly cleaned between trials and all trials were recorded using a closed-circuit camera mounted on the ceiling directly above the maze.

### Human Participants

Twenty-six participants were recruited from patient contact lists at Rush University Medical Center, Chicago, IL, United States. Participants either completed the mazes with visual cues (*n* = 10, mean chronological age = 22 years, *SD* = 3.84, mean verbal mental age = 6.26 years, *SD* = 3.02) or without visual cues (*n* = 15, mean chronological age = 24 years, *SD* = 4.9, mean verbal mental age = 7.57 years, *SD* = 1.29). The first group of participants provided data, which has not been previously published, on the paradigm with visual cues. The second group corresponds to participants tested in MacLeod et al. (2010) on the standard paradigm. All had a DNA-confirmed diagnosis of FXS. Informed consent was obtained from caregivers and assent was obtained from the individuals with FXS. All participants were paid $25 per hour for their participation in the study and were treated in accordance to the ethical principles established by the Research Ethics Board at the University of Ottawa. Both the ethics committees of the School of Psychology, University of Ottawa, and of the Rush University Medical Center approved the study. Written informed consent was obtained from carers of the participants. Participants also provided their written assent to participate in the study.

### Human Measures

A brief Medical History Questionnaire was administered to all caregivers of participants to screen for any problems that would exclude them from the study. Exclusion criteria were any significant health or vision difficulties (e.g., color blindness, amblyopia, astigmatism, etc.) that would impact controlling a joystick or viewing the maze stimuli. Four FXS participants were excluded from the study because they did not complete at least half of the mazes. These participants reported and exhibited symptoms of anxiety and appeared distracted throughout the administration.

### Human Apparatus

All participants were tested using a version of the virtual Hebb–Williams maze designed by Shore et al. (2001). Five mazes were eliminated from the original Hebb–Williams set for the purpose of this project because our pilot studies indicated that participants found these mazes too easy. In order to reduce administration time, a factor that is particularly important when testing participants affected by intellectual disability, only the most challenging mazes were used. All participants were tested on the remaining subset of mazes.

Experiments were performed on an Asus PC with a 19-inch Acer LCD monitor. Mazes were displayed at a resolution of 640 × 480 in full-screen mode. Participants navigated through the virtual environment at a constant velocity of 12 km/hr (forward, backward) and a turn rate of 50 degrees per second (left, right) using a Logitech Attack 3 joystick. Assuming a viewing height of 5 ft 6 in., the projection of the whole maze appeared to participants as 20 m^2^, and the diagonal straight line from start to finish was perceived as being located at a distance of 28.3 m.

**FIGURE 2:**
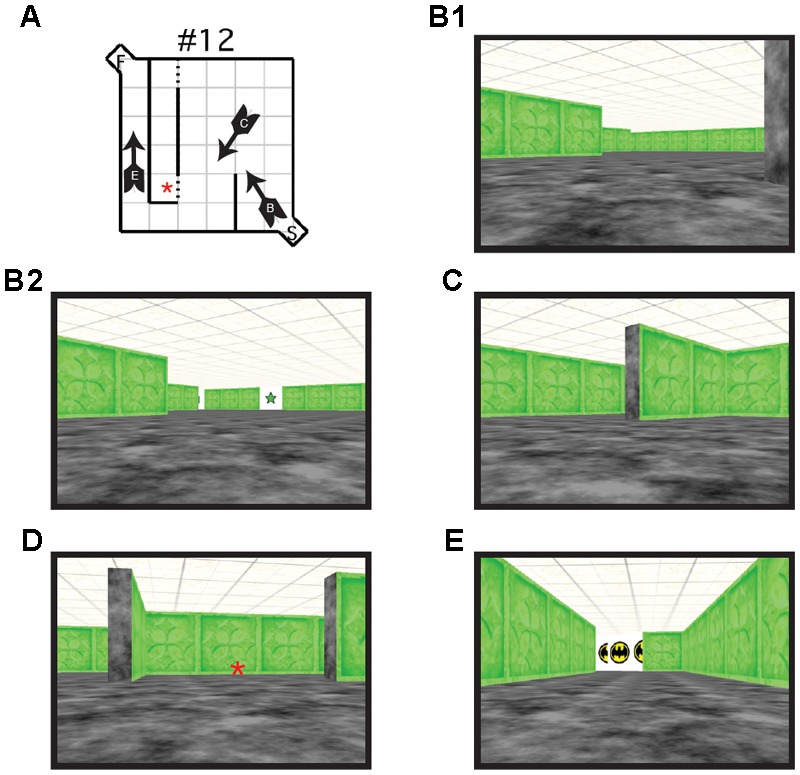
**(A)** Schematic illustration of virtual Hebb–Williams Maze #12. Arrows indicate the location and point of view of the navigator. Letters on the arrow correspond to the points of view illustrated in B, C, and E. An error was scored each time the participant crossed the threshold illustrated by the broken lines. **(B)** Scene from the start box of the maze without **(B1)** and with visual cues (**B2**, visible visual cue is a green 5-pointed star). **(C)** Scene illustrating a choice point leading to an error zone or to the correct escape path. **(D)** Scene illustrating an error zone (indicated by the red asterisk here and in **A**). **(E)** Scene illustrating the goal box.

Each maze was made up of a 6 × 6 room, with a 1 × 1 alcove at the entrance (start area) and exit (goal area) of the maze. Walls were created using textured rectangles that differed in color depending on the maze configuration. A different color was used for each maze configuration to indicate to participants that a new maze was being presented. The start alcove and the floors were textured with black and gray marble effect. Each wall of the goal alcove was white and contained the image of a comic book character to provide motivation and reward for the participants. The roof was textured using beige and brown mottled square tiles (**Figure 2**). For the visual cue group, the Hebb–Williams virtual maze was identical to that used in the standard condition, with the exception of the addition of visual cues. For each test maze, six images of simple geometric shapes (e.g., circle, square, and triangle) surrounded by a white background were added to the maze environment (10 cm × 10 cm). Geometric shapes were used because of they are easy to recognize and discriminate across age ranges. An identical set of six shapes was placed in each maze configuration. Each set was the same color as that of the test maze with the white background clearly distinguishing the shape from the maze wall (**Figure 1b**). Visual cue placement was the same as was used for the mouse apparatus.

### Human Procedure

All participants were individually tested by a research assistant, in a quiet room without their caregivers present. The tasks were administered during a 1–1.5 h session and presented in a standardized order as described above. Participants were trained on two types of practice mazes. An alley maze was presented first and enabled participants to establish how to adaptively maneuver through the virtual environment, while maintaining direct visual contact with the goal area. After meeting this criterion, a T-maze was presented in which participants had to choose a virtual navigational pathway in order to practice searching for the goal area of the maze. Visual cues were not provided during practice. Criterion was achieved in both acquisition mazes when participants could complete three consecutive maze trials in less than 30 s each. At any time if a participant exceeded 120 s during a trial, the trial was considered finished and the participant proceeded to the next maze. For both the acquisition and testing mazes, participants received a sticker as reward after each trial, and after completing all three trials of a maze they received a small piece of candy to be saved and consumed after the experiment was terminated.

After the acquisition sessions, human participants completed three trials of each test maze (**Figure 1b**). In between testing for each maze, participants were provided with a 2-min break, at which time a children’s DVD was played. After completing the fourth maze (#8) all participants were given a 10-min break. Latency for solving the maze (time taken from the maze entrance to exit) and number of errors (number of times a participant crossed a predefined error line – see **Figure 1b**) were calculated.

### Statistical Analyses

Because of floor effects and large variability across participants for both error and latency, data from Maze 11, the last maze tested, is excluded from this study. Outliers were removed from the data such that any score that was more than 2.5 SD away from the group mean was replaced by the next lowest or highest score that is within 2.5 SD of the mean. Because error and latency measures did not appear to meet the assumption of normality upon visual inspection, both measures were square root transformed.

#### Inverse Efficiency

Inverse efficiency allows for direct cross-species comparisons within the same statistical analysis (Shore et al., 2001). Moreover, inverse efficiency is the most appropriate measure for cross-species comparisons because humans and mice can adopt different strategies with respect to speed vs. accuracy to solve a maze (Shore et al., 2001). It is calculated by standardizing raw latency and raw error scores into *Z*-scores using the overall grand means and standard deviations from all subjects of the same species. Performance inverse efficiency scores were also calculated as follows: *Z*(Latency) + *Z*(Error)/2. Larger inverse efficiency scores indicate relatively poorer maze performance. This composite measure weights increases in latency and error equally and therefore accounts for differences between species in the relative contribution of errors and latency to overall performance (Shore et al., 2001).

#### Rate of Learning

To compare learning and memory across Species and Condition, we calculated a rate of learning measure using individual difference scores on inverse efficiency as follows: [(T1 - T2) + (T2 - T3)]/2. A positive value indicates that efficiency increased across trials.

#### Difficulty

Two indices of difficulty were computed: one for learning and one for performance on the first trial. For learning, difficulty was computed using the method described in Meunier et al. (1986). We examined the relative difficulty of each Maze for humans and mice and the Standard and With Visual Cue conditions separately. A difficulty index (*D*) was calculated as follows: mean number of errors across trials/number of error zones. The number of error zones were determined according to Rabinovitch and Rosvold (1951). We also computed an index of difficulty for performance on the first trial by modifying the computation proposed by Meunier et al. (1986). Difficulty was calculated as follows: mean number of errors for the first trial/number of error zones.

#### Analysis of Variance (ANOVAs)

All statistical analyses were conducted using SPSS. To examine the influence of adding a Visual Cue, a 2 × 2 × 6 × 3 mixed-design ANOVA was conducted on latency, error, and inverse efficiency with Species (Humans, Mice) and Condition (Standard, Visual Cue) as independent-groups variables and Maze (2, 4, 5, 8, 12, and 9) and Trial (1, 2, and 3) as repeated-measures variables. Note that whereas mice were tested on five trials, human participants were tested on three trials to cater to the limited attention span of affected individuals. To allow direct comparison of the two species within the same analysis, only trials one, two, and three were used from the mouse data. We did not analyze trials 4 and 5 because these additional trials may have engendered some additional fatigue/practice that might affect learning and that was not experienced by the human participants. Results are shown in **Tables 1–3**. The rate of learning variable was submitted to a 2 × 2 × 6 mixed-design ANOVA with Species (Humans, Mice) and Condition (Standard, Visual Cue) as independent-groups variables and Maze (2, 4, 5, 8, 9, and 12) as repeated-measures variable. Finally, we examined performance on Trial 1 only to compare the two species on visuo-spatial processing performance. A 2 × 6 mixed-design ANOVA with Species (Humans, Mice) as independent groups variable and Maze (2, 4, 5, 8, 12, and 9) as repeated-measures variable was conducted on the inverse efficiency measure for the Standard and Visual Cue conditions separately (**Table 4**). Because Maulchy’s test of sphericity was significant for most conditions, we applied the Greenhouse-Geisser correction to all effects involving repeated-measures factors.

**Table 1:** Analysis of variance (ANOVA) of latency between factors Species (humans and mice), Condition (standard and visual cues), Maze (2, 4, 5, 8, 9, and 12), and Trials (1, 2, and 3).

Test of between-subjects effects	*df*	*F*	$\eta_{p}^{2}$	*p*
Species	1	189.22	0.82	0.00
Visual Cue	1	1.12	0.03	0.30
Species ^∗^ Visual Cue	1	4.89	0.10	0.03
Error	43			
Trial	1.98	10.95	0.20	0.00
Trial ^∗^ Species	1.98	1.08	0.02	0.34
Trial ^∗^ Visual Cue	1.98	0.96	0.02	0.39
Trial ^∗^ Species ^∗^ Visual Cue	1.98	0.11	0.00	0.89
Error (Trial)	85.23			
Maze	3.76	24.49	0.36	0.00
Maze ^∗^ Species	3.76	10.06	0.19	0.00
Maze ^∗^ Visual Cue	3.76	3.44	0.07	0.01
Maze ^∗^ Species ^∗^ Visual Cue	3.76	3.37	0.07	0.01
Error (Maze)	161.52			
Trial ^∗^ Maze	7.54	1.19	0.03	0.31
Trial ^∗^ Maze ^∗^ Species	7.54	2.64	0.06	0.01
Trial ^∗^ Maze ^∗^ Visual Cue	7.54	0.39	0.01	0.92
Trial ^∗^ Maze ^∗^ Species ^∗^ Visual Cue	7.54	1.01	0.02	0.43
Error (Trial ^∗^ Maze)	324.16			

*df, degress of freedom; F, F statistic; $\eta_{p}^{2}$, partial eta squared; p, probability of a Type I error.*

**Table 2:** Analysis of variance (ANOVA) of errors between factors Species (humans and mice), Condition (standard and visual cues), Maze (2, 4, 5, 8, 9, and 12), and Trials (1, 2, and 3).

Test of between-subjects effects	*df*	*F*	$\eta_{p}^{2}$	*p*
Species	1	1.39	0.03	0.25
Visual Cue	1	1.01	0.02	0.32
Species ^∗^ Visual Cue	1	0.00	0.00	0.97
Error	41			
Maze	3.84	36.10	0.47	0.00
Maze ^∗^ Species	3.84	15.77	0.28	0.00
Maze ^∗^ Visual Cue	3.84	2.10	0.05	0.09
Maze ^∗^ Species ^∗^ Visual Cue	3.84	0.77	0.02	0.54
Error (Maze)	157.37			
Trial	1.97	13.25	0.24	0.00
Trial ^∗^ Species	1.97	0.43	0.01	0.65
Trial ^∗^ Visual Cue	1.97	0.07	0.00	0.93
Trial ^∗^ Species ^∗^ Visual Cue	1.97	1.35	0.03	0.27
Error (Trial)	80.64			
Maze ^∗^ Trial	7.32	1.07	0.03	0.39
Maze ^∗^ Trial ^∗^ Species	7.32	2.87	0.07	0.01
Maze ^∗^ Trial ^∗^ Visual Cue	7.32	0.50	0.01	0.84
Maze ^∗^ Trial ^∗^ Species ^∗^ Visual Cue	7.32	1.05	0.03	0.40
Error (Maze^∗^Trial)	300.07			

*df, degress of freedom; F, F statistic; $\eta_{p}^{2}$, partial eta squared; p, probability of a Type I error.*

**Table 3:** Analysis of variance (ANOVA) of inverse efficiency between factors Species (humans and mice), Condition (standard and visual cues), Maze (2, 4, 5, 8, 9, and 12), and Trials (1, 2, and 3).

Test of between-subjects effects	*df*	*F*	$\eta_{p}^{2}$	*p*
Species	1	0.12	0.00	0.73
Visual Cue	1	4.05	0.09	0.05
Species ^∗^ Visual Cue	1	5.09	0.11	0.03
Error	41			
Maze	3.11	36.30	0.47	0.00
Maze ^∗^ Species	3.11	13.43	0.25	0.00
Maze ^∗^ Visual Cue	3.11	4.83	0.11	0.00
Maze ^∗^ Species ^∗^ Visual Cue	3.11	3.73	0.08	0.01
Error (Maze)	127.59			
Trial	1.90	15.69	0.28	0.00
Trial ^∗^ Species	1.90	1.91	0.04	0.16
Trial ^∗^ Visual Cue	1.90	1.36	0.03	0.26
Trial ^∗^ Species ^∗^ Visual Cue	1.90	0.89	0.02	0.41
Error (Trial)	78.02			
Maze ^∗^ Trial	5.90	1.77	0.04	0.11
Maze ^∗^ Trial ^∗^ Species	5.90	4.34	0.10	0.00
Maze ^∗^ Trial ^∗^ Visual Cue	5.90	0.47	0.01	0.83
Maze ^∗^ Trial ^∗^ Species ^∗^ Visual Cue	5.90	1.17	0.03	0.32
Error (Maze^∗^Trial)	241.78			

*df, degress of freedom; F, F statistic; $\eta_{p}^{2}$, partial eta squared; p, probability of a Type I error.*

**Table 4:** Analysis of variance (ANOVA) of rate of learning between factors Species (humans and mice), Condition (standard and visual cues), Maze (2, 4, 5, 8, 9, and 12).

	*df*	*F*	$\eta_{p}^{2}$	*p*
Species	1	3.10	0.07	0.09
Visual Cue	1	1.73	0.04	0.20
Species ^∗^ Visual Cue	1	0.30	0.01	0.59
Error	41			
Maze	4.08	2.35	0.05	0.06
Maze ^∗^ Species	4.08	2.32	0.05	0.06
Maze ^∗^ Visual Cue	4.08	0.70	0.02	0.59
Maze ^∗^ Species ^∗^ Visual Cue	4.08	0.84	0.02	0.50
Error (Maze)	167.18			

*df, degress of freedom; F, F statistic; $\eta_{p}^{2}$, partial eta squared; p, probability of a Type I error.*

Alpha adjustment was not performed because is was deemed too conservative on the grounds that a valid paradigm is likely to yield non-significant differences between species and because of the potentially large number of mean comparisons following significant interactions. Instead, we focused on effect sizes for comparing relevant means operationalized as follows: no effect: Cohen’s *d* of 0.0–0.2; small effect: Cohen’s *d* of 0.2–0.5; medium effect: Cohen’s *d* of 0.5–0.8; large effect: Cohen’s *d* of 0.8 and more.

## Results

### Latency

As expected, the main effect of Trial was significant with latency decreasing from Trial 1 (*M* = 5.13; *SE* = 0.12) to Trial 2 (*M* = 4.75; *SE* = 0.13) to Trial 3 (*M* = 4.53; *SE* = 0.11), indicating that learning occurred whereby participants took progressively less time to complete a maze from Trial 1 to Trial 3. We focus on effects involving a Species × Condition interaction since significant findings involving this interaction suggest that the two species react differently to the presence of visual cues. The Species × Condition × Maze interaction was significant. For humans, comparing the Standard Condition to the Visual Cue Condition for each maze, participants took less time to find the goal with the Visual Cue for mazes 4 (*d* = -0.4), 5 (*d* = -0.6), and 9 (*d* = -0.7). Participants took more time to find the goal with the Visual Cue for mazes 8 (*d* = 0.2), 12 (*d* = 0.3). There was no effect of Visual Cue for maze 2 (*d* = 0.0). Mice took more time finding the goal with the Visual Cue for mazes 4 (*d* = 0.3), 5 (*d* = 0.4), 8 (*d* = 0.4), and 9 (*d* = 0.7). There was no effect of Visual Cue for mazes 2 (*d* = 0.0) and 12 (*d* = 0.1). Hence, there was consistency between the two species only for Maze 2 where the Visual Cue did not improve the speed at which the maze was solved for both humans and mice.

### Errors

As expected, the main effect of Trial was significant with number of errors decreasing from Trial 1 (*M* = 1.65; *SE* = 0.06) to Trial 2 (*M* = 1.43; *SE* = 0.05) to Trial 3 (*M* = 1.31; *SE* = 0.05), indicating that learning occurred whereby participants made progressively fewer errors from Trial 1 to Trial 3. The Species × Condition Interaction was not significant, nor were any of the interactions involving the Species × Condition effect. The main effect of Condition was not significant. These results suggest that both species reacted similarly to the Visual Cue whereby adding a Visual Cue did not influence errors committed while solving the maze for both humans and mice.

### Inverse Efficiency

**FIGURE 3:**
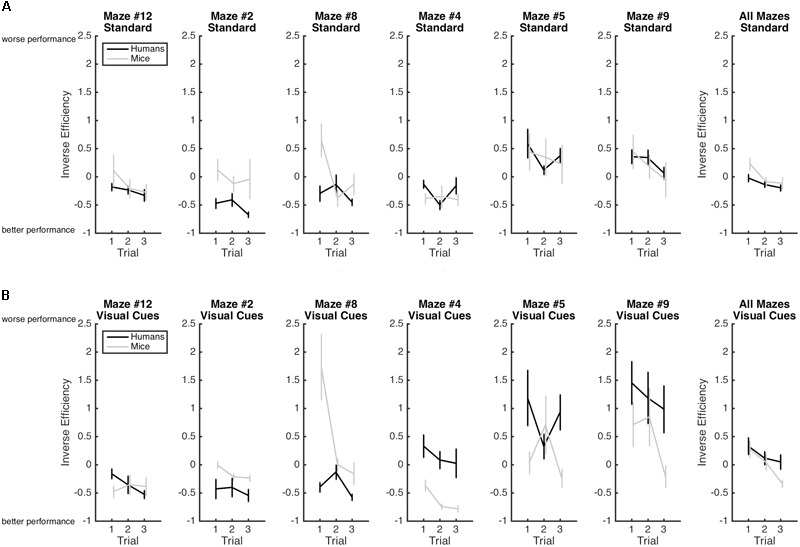
Mean inverse efficiency and standard-errors for both humans (black) and mice (gray) in the standard Hebb–Williams maze paradigm **(A)** and in the condition with visual cues **(B)**. Larger inverse efficiency scores indicate less efficient maze-solving performance.

Results are illustrated in **Figure 3**. As expected the main effect of Trial was significant with efficiency increasing from Trial 1 (*M* = 0.20; *SE* = 0.05), to Trial 2 (*M* = -0.02; *SE* = 0.05), to Trial 3 (*M* = -0.15; *SE* = 0.05), indicating again that learning occurred whereby participants became progressively more efficient at solving the mazes from Trial 1 to Trial 3. This improvement in performance is best captured by the Rate of Learning analyses presented below. The Species × Condition Interaction was significant. The Species × Condition × Maze interaction was also significant. For humans, comparing the Standard Condition to the Visual Cue Condition for each maze, performance was more efficient with the Visual Cue for Maze 2 (*d* = -0.2), 4 (*d* = -0.7), 5 (*d* = -0.5), and 9 (*d* = -1.0). Performance was less efficient with the Visual Cue for Mazes 12 (*d* = 0.2), and there was no effect of Condition for maze 8 (*d* = 0.1). For mice, performance was more efficient with the Visual Cue for Mazes 12 (*d* = -0.8), 2 (*d* = -0.6), 4 (*d* = -0.4), 5 (*d* = -0.2). Performance was less efficient with Visual Cue for Maze 8 (*d* = 0.8) and 9 (*d* = 0.8). Hence, for both species, the addition of a visual cue improved efficiency for mazes 2, 4, 5 but not for the other mazes.

Considering that comparing the Standard with the Visual Cue conditions yielded inconsistent results, we also compared humans and mice on each maze for the Standard and Visual Cue conditions separately. For the Standard condition, Maze 4 (*d* = 0.4), Maze 5 (*d* = 0.0), Maze 9 (*d* = 0.0), and Maze 12 (*d* = -0.4) yielded no or small species differences. Maze 2 (*d* = -1.4) and Maze 8 (*d* = -1) yielded large species differences. For the Visual Cue condition, only Maze 12 (*d* = 0.2) yielded a small species difference. The differences between humans and mice were large for Maze 2 (*d* = -0.7), Maze 4 (*d* = 1.3), Maze 5 (*d* = 0.7), Maze 8 (*d* = -2.2), and Maze 9 (*d* = 0.7). Hence, performance was generally consistent across species for the Standard condition but not for the Visual Cue condition.

For performance on the first trial only, we report findings with respect to the Species variable only. For the Standard Condition, the main effect of Species was significant [*F*(1,24) = 4.28, *p* = 0.05, $\eta_{p}^{2}$ = 0.15]. The Maze × Species interaction was also significant [*F*(3.65,87.33) = 2.77, *p* = 0.04, $\eta_{p}^{2}$= 0.10]. For the Visual Cue Condition, the main effect of Species was not significant [*F*(1,24) < 1]. The Maze × Species interaction was significant [*F*(2.88,48.96) = 8.17, *p* < 0.01, $\eta_{p}^{2}$ = 0.33]. To explore these significant interactions, we compared humans and mice on each maze for the Standard and Visual Cue conditions separately. For the Standard condition, Maze 5 (*d* = 0.4) and Maze 9 (*d* = -0.2) yielded small species differences. Maze 4 (*d* = 0.7) and Maze 12 (*d* = -0.7) yielded medium species differences. Maze 2 (*d* = -1.7) and Maze 8 (*d* = -2.7) yielded large species differences. For the Visual Cue condition, Maze 9 (*d* = 0.6) yielded medium species differences. All other mazes yielded large species differences (Maze 2: *d* = -0.8; Maze 4: *d* = 1.1; Maze 5: *d* = 0.9; Maze 8: *d* = -4.4; Maze 12: *d* = 0.8). These results suggest that performance obtained on Mazes 5 and 9 of the standard paradigm provide the best measure of visuo-spatial processing and problem solving across species.

### Rate of Learning

**FIGURE 4:**
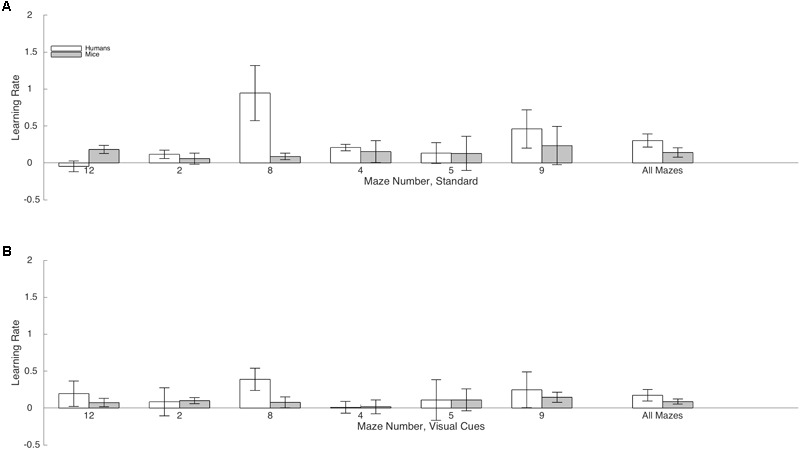
Mean rate of learning and standard-errors for both humans (white) and mice (gray) in the standard Hebb–Williams maze paradigm **(A)** and in the condition with visual cues **(B)**. A positive value indicates that participants became more efficient at solving the maze from trials 1 to 2 and from trials 2 to 3 (averaged). A value of zero indicates no learning.

Rate of Learning indicates the amount by which efficiency increased across trials. Results are illustrated in **Figure 4**. The Species × Condition interaction was not significant. The Species × Condition × Maze interaction was significant. For humans, comparing the Standard Condition to the Visual Cue Condition for each maze, rate of learning was superior with the Visual Cue for Maze 4 (*d* = -0.4), Maze 5 (*d* = -0.3), Maze 8 (*d* = -0.3), Maze 9 (*d* = -0.2), and Maze 12 (*d* = -0.6). Rate of learning was inferior with the Visual Cue for Maze 2 (*d* = 0.2). For mice, rate of learning was superior with the Visual Cue for Maze 12 (*d* = -0.2). Rate of learning was inferior with the Visual Cue for Mazes 8 (*d* = 0.2), 4 (*d* = 1), and 9 (*d* = 0.2). There was no effect of Condition for Mazes 2 (*d* = 0.1) and 5 (*d* = 0.1). Except for Maze 12 for the humans and Maze 4 for the mice, effect sizes were generally small in both species, suggesting that adding a Visual Cue had very little impact on Rate of Learning for both humans and mice.

We also compared humans and mice on each maze for the Standard and Visual Cue conditions separately. For the Standard condition Maze 2 (*d* = 0.04), Maze 4 (*d* = 0.02) and Maze 5 (*d* = 0.00), Maze 9 (*d* = -0.17) and Maze 12 (*d* = -0.28), yielded no or small species differences. Differences between humans and mice were medium for Maze 8 (*d* = -0.77). For the Visual Cue condition, Maze 2 (*d* = -0.42), Maze 4 (*d* = -0.17), Maze 5 (*d* = -0.01), and Maze 9 (*d* = -0.28) yielded no or small species differences. Differences between humans and mice were large for Maze 8 (*d* = -1.02) and Maze 12 (*d* = 1.15). Hence, performance was generally consistent across species for both the Standard and the Visual Cue condition for three out of the six mazes tested (2, 4, and 5).

### Difficulty

Difficulty was computed for each maze for humans and mice and for the standard and the visual cue conditions separately for performance across trials and for performance on the first trial. Mazes were then ordered in ascending levels of difficulty to examine similarities/differences in the pattern of performance across the two species. For performance across trials (**Table 5**), while patterns of difficulty were not identical across species some consistencies were observed. Mazes 2 and 4 were among the three easiest mazes and Mazes 5 and 12 were among the three hardest mazes for both humans and mice. Moreover, for each species, mazes 5, 9, and 12 were most difficult and mazes 2, 4, and 8, were easiest irrespective the presence of visual cues. For performance on the first trial (**Table 6**), Mazes 2 and 4 were among the three easiest and Maze 5 was among the three most difficult for both species irrespective of visual cues.

**Table 5:** Relative difficulty (D) of the different mazes across trials for the Standard and Visual Cue conditions for Humans and Mice separately.

Humans				Mice			
Standard		Cue		Standard		Cue	
Maze	*D*	Maze	*D*	Maze	*D*	Maze	*D*
2	0.21	2	0.27	4	0.26	4	0.34
8	0.41	8	0.48	9	0.52	9	0.38
4	0.44	4	0.66	2	0.58	2	0.57
9	0.61	12	0.73	5	0.9	8	0.81
5	1.08	9	0.91	12	1.1	5	0.92
12	1.12	5	1.34	8	1.23	12	1.67

*Results are presented in ascending order with the higher the D index, the more difficult the maze. Details are provided in the text.*

**Table 6:** Relative difficulty (D) of the different mazes for performance on the first trial for the Standard and Visual Cue conditions for Humans and Mice separately.

Humans				Mice			
Standard		Cue		Standard		Cue	
Maze	*D*	Maze	*D*	Maze	*D*	Maze	*D*
4	0.00	2	0.28	4	0.35	4	0.41
2	0.29	8	0.47	2	0.62	2	0.61
8	0.38	4	0.86	9	0.87	12	0.75
9	0.94	12	1.44	5	0.91	5	0.75
5	1.27	5	1.67	8	1.29	9	1.00
12	1.47	9	1.68	12	2.00	8	2.15

*Results are presented in ascending order with the higher the D index, the more difficult the maze. Details are provided in the text.*

### Activity Level in Mice

Previous studies suggest that FXS KO mice may exhibit increased activity levels as compared to wild type mice (e.g., Mineur et al., 2002). Thus, we assessed activity levels by obtaining a count of the number of line crosses per unit of time for trial 1 of maze 12. The latter maze was chosen because it has the least number of partitions thus allowing for the clearest observation of locomotion. We restricted our analysis to trial 1 because performance on this trial is independent of learning and memory and reflects exploratory behavior. An independent samples *t*-test revealed no significant differences in activity levels between the group tested in the standard paradigm and the group tested with visual cues.

## Discussion

Using FXS as a model disease, we examined the potential utility of the Hebb–Williams maze paradigm (Hebb and Williams, 1946; Rabinovitch and Rosvold, 1951) for translational and drug discovery research on the basis of three validation criteria. The paradigm allows direct comparison of humans and animals on tasks that are behaviorally equivalent (criterion #1) (Shore et al., 2001; see also Gabel et al., 2016) and measures visuo-spatial abilities, a cognitive domain for which FXS individuals and KO mice show impairments as compared to their respective control groups (condition #2) (e.g., Crowe and Hay, 1990; Cornish et al., 1998, 1999; Kogan et al., 2004a). We compared the performance of affected humans and mice across multiple conditions and measures to evaluate whether the paradigm engages comparable cognitive mechanisms in both species (criterion #3). These cross-species comparisons allowed us to identify which conditions, if any, produce comparable patterns of performance across species and therefore offer the best potential for extrapolating results from mice to humans (Willner, 1986; Shore et al., 2001; van der Staay et al., 2010; Sjoberg, 2017). The discussion of our results focuses on measures of performance that allow direct cross-species comparisons, namely efficiency, rate of learning, and difficulty.

We compared performance of FXS humans and KO mice on the standard paradigm as well as on a variation of the paradigm where visual cues were added to the mazes. Our goal was to examine whether this variable has a comparable impact on humans and mice, which would provide support for the notion that the paradigm taps into comparable underlying cognitive mechanisms in both species (Willner, 1986; Shore et al., 2001; van der Staay et al., 2010; Sjoberg, 2017). Our results provide mixed evidence regarding the influence of this manipulation. Specifically, the addition of a visual cue improved performance efficiency for both species for three out of six mazes (i.e., 2, 4, and 5). In contrast, rate of learning was enhanced by the presence of visual cues for both species for only one maze (i.e., 12). Finally, looking at the human and mice data separately, we find that relative maze difficult was comparable with and without the presence of visual cues, either for difficulty in learning the maze across trials, or for performance on the first trial. Taken together, these results suggest that visual cues do not consistently improve spatial information processing in FXS with the exception of specific mazes where results were consistent across species (information pertaining to specific mazes is presented below). While visual cues have been shown to improve spatial learning performance in typically developing human adults and mice (reviewed by Chan et al., 2012), this is not the case for other populations. Consistent with our findings, there is evidence that visual cues do not improve spatial learning in individuals with ADHD (Robaey et al., 2016), a diagnosis that is commonly comorbid with FXS (Sullivan et al., 2006). FXS is also highly co-morbid with ASD (Hatton et al., 2006) and in this population, the literature on the influence of visual cues on spatial learning is inconclusive, in part because comparisons are often made between conditions that differ with respect to many variables (reviewed by Smith, 2015). Looking at the general effect of landmarks on navigation performance, and seemingly in agreement with our findings, it appears that impairments in navigation are not diminished by the presence of visual cues in participants with ASD (Lind et al., 2013).

Because there is considerable variation in nomenclature and interpretation of results involving visual cues, it is difficult to provide a simple cohesive interpretation of our findings. The presence of visual cues is thought to shift the strategies implicated in solving mazes from spatial learning toward response-based learning (e.g., Packard and McGaugh, 1996). Response-based strategies rely on reinforcement of stimulus-response contingencies, allowing participants to solve mazes by learning that they can reach the goal by making a specific body turn at a choice point in the maze. Because humans affected by FXS have been shown to display perseverative behaviors (Van Dam et al., 2000; Kogan et al., 2009), and because we used the same cues across mazes, it is possible that learning of stimulus-response contingencies in the earlier mazes interfered with learning new stimulus-response contingencies in the later mazes, which would have rendered the visual cues ineffective with time. To explore this possibility, we conducted separate analyses to examine Species × Condition interactions for the first maze tested and for the last maze tested on inverse efficiency. In partial agreement with this interpretation, the interaction was not significant for the first maze but approached significance for the last maze (first maze: *F* = 0.827, *p* = 0.368, ns; last maze: *F* = 3.702, *p* = 0.061). However, inspection of **Figure 4** suggests that rate of learning was not linked to testing order for either condition. Another possible explanation for our results is that participants may have had difficulty discriminating between cues that provided information about relative position and those that could be used for a turning response. Indeed, while the visual cues were informative because they were fixed and therefore could indicate to the participant their location in the maze, they were not all located at decision points in the mazes. Additional studies are needed to resolve these discrepancies and to confirm under which conditions visual cues should be used in this paradigm for cross-species extrapolation. Future research should include probe trials where visual cues are removed after a maze has been solved to determine whether they contributed to improvements across trials (e.g., Moffat and Resnick, 2002; Vorhees and Williams, 2014). Moreover, distinct visual cues should be used across different mazes to avoid the possibility of perseveration of responding to similar cues from one maze to the next. Finally, because there is lower reliance on the visual modality for navigation in mice (Brown and Wong, 2007), it would be interesting to examine whether using cues that are optimal for each species (e.g., olfactory in mice, visual in humans) at decision points in a maze generates more comparable findings across species^3^.

It should be noted that humans and mice displayed more consistent results with respect to the influence of visual cues for measures that incorporate errors (error, inverse efficiency, rate of learning) than for the measure of latency. Pollard and Lysons (1969) have suggested that measures based on errors are more indicative of learning and memory, whereas those based on time are more indicative of exploratory and motivational factors. Hence, it is possible that humans and mice reacted similarly to visual cues with respect to learning as indexed by measures of error, but not with respect some of the other behaviors triggered by the maze environment (i.e., those indexed by latency, including efficiency). Gandhi et al. (2014a) also found that it was the measure of errors that was sensitive to the effects of the mGluR antagonist MPEP, which concomitantly reversed the deficits in PSD-95 reactivity to Hebb–Williams maze learning. These data suggest that future studies focusing on molecular pathways mediated by FMRP (Fragile × Mental Retardation protein) and involved in synaptic plasticity should include errors as a dependent variable to evaluate the effect of pharmacological, genetic, or other manipulations.

Cross-species comparisons revealed comparable patterns of performance for FXS humans and mice for some mazes but not others. Focusing on the measure of efficiency, which reflects overall performance on the mazes by combining error rates and latency, four mazes (4, 5, 9, and 12) produced consistent results in humans and mice in the standard condition and one maze (12) in the visual cue condition. For rate of learning, which reflects improvements in performance across trials, three out of the six mazes tested (i.e., 2, 4, and 5) produced consistent results for humans and mice for both conditions. We also conducted cross-species comparisons by measuring the level of difficulty of each maze for each species and each condition separately. The relative difficulty of four mazes (2, 4, 5, and 12) was consistent in humans and mice, irrespective of the presence of visual cues. Finally, we compared efficiency and difficulty across species and for each condition separately for performance on the first trial only. This allowed us to distinguish the effects of learning/memory across trials from the ability to utilize visual information to solve a novel spatial task on the first trial (Hebb and Williams, 1946). Only two mazes (i.e., 5 and 9) tested in the standard condition yielded comparable results for humans and mice. These two mazes also produced consistent results in humans and mice in terms of difficulty for the visual cue condition. At the time of this writing, we retrieved only two studies that have directly compared humans and mice on the Hebb–Williams maze paradigm (Shore et al., 2001; Gabel et al., 2016). These studies also report heterogeneous cross-species consistencies across mazes tested in typically developing participants. Shore et al. (2001) report consistent efficiency and rate of learning for three mazes (6, 8, and 12), however, statistical results for these group comparisons are not provided. Gabel et al. (2016) report consistent efficiency and rate of learning for all mazes tested (5, 6, 11, and 12). The discrepancy between our results and those obtained with typically developing participants underline the relevance of directly comparing affected humans and mice to evaluate the validity of translational paradigms in FXS.

To provide practical advice regarding which conditions and mazes should be used for extrapolating results from mice to humans in translational research, we examined the overall pattern of consistencies across the different measures and identified those that provided at least two equivalent cross-species comparisons. Irrespective of visual cues, Mazes 2, 4, and 5 provided no or small species differences for at least two measures of performance, suggesting that these conditions have good potential to measure spatial learning/memory across species. With regard to performance on the first trial, which reflects visuo-spatial problem solving, Mazes 5 and 9 without visual cues produced consistent results.

Notwithstanding the aforementioned cross-species consistencies, many conditions failed to produce comparable results in humans and mice, which highlights the need for additional research focusing on the nature of the cognitive processes implicated in this paradigm. This desideratum is not merely theoretical but has very practical implications to clinical researchers submitting their rationale and predictions about variables that will change in drug trials to the drug regulatory bodies (e.g., FDA in the United States). Specific outcomes have not been scaled from mice to humans in recent trials such as the trial of Arbaclofen in FXS, which was intended to improve social avoidance (Berry-Kravis et al., 2017). One important obstacle to demonstrating efficacy may have been the lack of a translational measure validated on the basis of criteria such as those specified in the present article. Within this context, it would be particularly important to further investigate the purported dissociation between the cognitive mechanisms underlying performance on the first trial vs. improvements in performance across trials. Indeed, there were no differences between FXS participants and controls for performance on the first trial in MacLeod et al. (2010). In contrast, deficits in spatial learning have been reported using both the Hebb–Williams maze paradigm (MacLeod et al., 2010) as well as other maze paradigms (e.g., radial maze: Mineur et al., 2002; cross shaped maze: Dobkin et al., 2000). If our findings of comparable and intact performance on the first trial vs. comparable and impaired learning across trials were corroborated, then it would support the utility of these two measures to target distinct cognitive functions in drug efficacy trials.

Finally, differences between mice and humans may have arisen from the use of a virtual environment with humans. Indeed, virtual navigation only assesses visually-based learning without input from the other modalities recruited when participants are actually moving through a real space (e.g., proprioception, motor). Despite these differences, studies in the elderly generally indicate that more often than not, results with virtual mazes generalize to real maze paradigms (Moffat, 2009). Whether this is also the case in individuals affected by FXS has yet to be determined.

As whole, our results support the potential utility and validity of the Hebb–Williams maze paradigm for measuring visual-spatial abilities in translational research in FXS. First, it is the only paradigm that has shown comparable patterns of results in humans and mice using both the homology of impairments approach (MacLeod et al., 2010) and the direct comparison approach described here. Second, the paradigm measures visuo-spatial problem solving as well as spatial learning and memory, two processes that have been shown to be impacted by the lack of FMRP and important targets for treatment (Cornish et al., 1999). Third, while more work is needed in this area, there is evidence that performance on the Hebb–Williams maze paradigm can be examined and related at multiple levels of analysis including cognitive and behavioral functioning (e.g., MacLeod et al., 2010), anatomical pathways (e.g., Hunsaker et al., 2008), and molecular pathways (e.g., Gandhi et al.). In light of these promising results, we feel that increased emphasis needs to be directed toward specifying the practical parameters for the Hebb–Williams paradigm as well as other paradigms that allow direct comparison of humans and animals (e.g., object-discrimination learning and reversal, radial mazes, see Boutet et al., 2007; Kogan et al., 2009; Gilmour et al., 2013; McGonigle and Ruggeri, 2014; Gabel et al., 2016; see also Watase and Zoghbi, 2003). These efforts are critically important to extrapolating results of drug discovery as well as basic cellular and molecular research from animal studies to humans and therefore in ultimately improving the lives of those affected by FXS.

## Author Contributions

IB drafted the manuscript and contributed to the conceptualization and data analyses. CC contributed to the conceptualization and methodological design for the human Hebb–Williams Mazes, as well as aiding in analysis. He was available for consultation and reviewed the manuscript. LM contributed to the conceptualization and design of the experiments described, as well as tested participants as part of her dissertation research. CM contributed to the conceptualization, methodological design, and data collection for the animal testing. He also provided the necessary laboratory space to conduct testing. MH provided guidance on the conceptualization of the paradigm with visual cues and reviewed the manuscript. EB-K assisted with the conceptualization of the project and was responsible for the recruitment of participants at RUSH University Medical Center. She also reviewed the manuscript and provided feedback. RG contributed to the behavioral testing of mice. CK is lab director. He conceptualized the research project, obtained funding, and provided guidance and assistance in all phases of the project and reviewed and revised the manuscript.

## Conflict of Interest Statement

The authors declare that the research was conducted in the absence of any commercial or financial relationships that could be construed as a potential conflict of interest.

## Footnotes

1 http://www.ncbi.nlm.nih.gov/omim

2 Note that criterion #3 is derived from Willner (1986) and Sjoberg (2017). While Willner’s discussion of validity focuses on the cognitive processes that underlie psychopathology (e.g., memory, perception, motivation, etc.), Sjoberg extends the discussion to include underlying biological mechanisms. While both are relevant and important in FXS, the present study, which was conducted from an experimental psychology perspective, focuses on the cognitive functions measured by the Hebb–Williams paradigm, namely visuo-spatial abilities.

3 Using different navigational cues across species might have implications for criterion #1. However, one must consider all criteria when making methodological decisions in translational research. If presenting each specie with tasks that are more ecologically relevant leads to the measurement of more comparable underlying functions (criterion 3), than we would consider this more important than using tasks that are identical across species (see Willner, 1986, for a discussion of face vs. construct validity in animal models).

## References

[B1] Andersen R. Buneo C. (2002). Intentional maps in posterior parietal cortex. *Annu. Rev. Neurosci.* 25 189–220. 10.1146/annurev.neuro.25.112701.142922 12052908

[B2] Backes M. Genc B. Schreck J. Doerfler W. Lehmkuhl G. (2002). Cognitive and behavioural profile of fragile X boys: correlations to molecular data. *Am. J. Med. Genet.* 95 150–156. 10.1002/1096-8628(20001113)95:2<150::AID-AJMG11>3.0.CO;2-1 11078566

[B3] Baker K. B. Wray S. P. Ritter R. Mason S. Lanthorn T. H. Savelieva K. V. (2010). Male and female Fmr1 knockout mice on C57 albino background exhibit spatial learning and memory impairments. *Genes Brain Behav.* 9 562–574. 10.1111/j.1601-183X.2010.00585.x 20398059

[B4] Baumgardner T. Reiss A. Freund L. Abrams M. (1995). Specification of the neurobehavioral phenotype in males with fragile X syndrome. *Pediatrics* 95 744–752. 7724315

[B5] Berry-Kravis E. Hagerman R. Visootsak J. Budimirovic D. Kaufmann W. E. Cherubini M. (2017). Arbaclofen in fragile X syndrome: results of phase 3 trials. *J. Neurodev. Disord.* 12:3. 10.1186/s11689-016-9181-6 28616094 PMC5467054

[B6] Berry-Kravis E. Hessl D. Coffey S. Hervey C. Schneider A. Yuhas J. (2009). A pilot open label, single dose trial of fenobam in adults with fragile X syndrome. *J. Med. Genet.* 46 266–271. 10.1136/jmg.2008.063701 19126569 PMC2658751

[B7] Berry-Kravis E. Knox A. Hervey C. (2011). Targeted treatments for fragile X syndrome. *J. Neurodev. Disord.* 3 193–210. 10.1007/s11689-011-9074-7 21484200 PMC3261278

[B8] Berry-Kravis E. Krause S. E. Block S. S. Guter S. Wuu J. Leurgans S. (2006). Effect of CX516, an AMPA-modulating compound, on cognition and behavior in fragile X syndrome: a controlled trial. *J. Child Adolesc. Psychopharmacol.* 16 525–540. 10.1089/cap.2006.16.525 17069542

[B9] Bostrom C. Yau S. Y. Majaess N. Vetrici M. Gil-Mohapel J. Christie B. R. (2016). Hippocampal dysfunction and cognitive impairment in Fragile-X Syndrome. *Neurosci. Biobehav. Rev.* 68 563–574. 10.1016/j.neubiorev.2016.06.033 27345143

[B10] Boutet I. Milgram N. W. Freedman M. (2007). Cognitive decline and human (Homo sapiens) aging: an investigation using a comparative neuropsychological approach. *J. Comp. Psychol.* 121 270–281. 10.1037/0735-7036.121.3.270 17696653

[B11] Braun K. Segal M. (2000). FMRP involvement in formation of synapses among cultured hippocampal neurons. *Cereb. Cortex* 10 1045–1052. 10.1093/cercor/10.10.1045 11007555

[B12] Brown R. E. Wong A. A. (2007). The influence of visual ability on learning and memory performance in 13 strains of mice. *Learn. Mem.* 14 134–144. 10.1101/lm.473907 17351136 PMC1838554

[B13] Chan E. Baumann O. Bellgrove M. A. Mattingley J. B. (2012). From objects to landmarks: the function of visual location information in spatial navigation. *Front. Psychol.* 3:304. 10.3389/fpsyg.2012.00304 22969737 PMC3427909

[B14] Chen L. Toth M. (2001). Fragile X mice develop sensory hyperreactivity to auditory stimuli. *Neuroscience* 103 1043–1050. 10.1016/S0306-4522(01)00036-7 11301211

[B15] Cornish K. Munir F. Cross G. (1998). The nature of the spatial deficit in young females with Fragile-X syndrome: a neuropsychological and molecular perspective. *Neuropsychologia* 36 1239–1246. 10.1016/S0028-3932(97)00162-0 9842768

[B16] Cornish K. M. Munir F. Cross G. (1999). Spatial cognition in males with Fragile-X syndrome: evidence for a neuropsychological phenotype. *Cortex* 35 263–271. 10.1016/S0010-9452(08)70799-8 10369098

[B17] Crowe S. F. Hay D. A. (1990). Neuropsychological dimensions of the fragile X syndrome: support for a non-dominant hemisphere dysfunction hypothesis. *Neuropsychologia* 28 9–16. 10.1016/0028-3932(90)90082-Y 2138257

[B18] Dobkin C. Rabe A. Dumas R. El Idrissi A. Haubenstock H. Ted Brown W. (2000). Fmr1 knockout mouse has a distinctive strain-specific learning impairment. *Neuroscience* 100 423–429. 10.1016/S0306-4522(00)00292-X 11008180

[B19] Errijgers V. Kooy R. F. (2004). Genetic modifiers in mice: the example of the fragile X mouse model. *Cytogenet. Genome Res.* 105 448–454. 10.1159/000078218 15237233

[B20] Farzin F. Perry H. Hessl D. Loesch D. Cohen J. Bacalman S. (2006). Autism spectrum disorders and attention-deficit/hyperactivity disorder in boys with the fragile X premutation. *J. Dev. Behav. Pediatr.* 27 S137–S144. 10.1097/00004703-200604002-00012 16685180

[B21] Fisch G. S. Hao H. K. Bakker C. Oostra B. A. (1999). Learning and memory in the FMR1 knockout mouse. *Am. J. Med. Genet.* 84 277–282. 10.1002/(SICI)1096-8628(19990528)84:3<277::AID-AJMG22>3.0.CO;2-W 10331607

[B22] Frankland P. W. Wang Y. Rosner B. Shimizu T. Balleine B. W. Dykens E. M. (2004). Sensorimotor gating abnormalities in young males with fragile X syndrome and Fmr1-knockout mice. *Mol. Psychiatry* 9 417–425. 10.1038/sj.mp.4001432 14981523

[B23] Gabel L. A. Manglani M. Escalona N. Cysner J. Hamilton R. Pfaffmann J. (2016). Translating dyslexia across species. *Ann. Dyslexia* 66 319–336. 10.1007/s11881-016-0125-3 27013331

[B24] Gandhi R. M. Kogan C. S. Messier C. MacLeod L. S. (2014b). Visual–spatial learning impairments are associated with hippocampal PSD-95 protein dysregulation in a mouse model of fragile X syndrome. *Neuroreport* 25 255–261. 10.1097/WNR.0000000000000087 24323121 PMC3925173

[B25] Gandhi R. M. Kogan C. S. Messier C. (2014a). 2-Methyl-6-(phenylethynyl) pyridine (MPEP) reverses maze learning and PSD-95 deficits in Fmr1 knock-out mice. *Front. Cell. Neurosci.* 8:70. 10.3389/fncel.2014.00070 24701200 PMC3965849

[B26] Gilmour G. Arguello A. Bari A. Brown V. J. Carter C. Floresco S. B. (2013). Measuring the construct of executive control in schizophrenia: defining and validating translational animal paradigms for discovery research. *Neurosci. Biobehav. Rev.* 37(9 Pt B) 2125–2140. 10.1016/j.neubiorev.2012.04.006 22548905

[B27] Grossman A. Elisseou N. McKinney B. Greenough W. (2006). Hippocampal pyramidal cells in adult *Fmr1* knockout mice exhibit an immature-appearing profile of dendritic spines. *Brain Res.* 1084 158–164. 10.1016/j.brainres.2006.02.044 16574084

[B28] Hagerman R. J. (1987). Fragile X syndrome. *Curr. Probl. Pediatr.* 17 626–674. 10.1016/0045-9380(87)90011-9 3325231

[B29] Hanlon F. M. Weisend M. P. Hamilton D. A. Jones A. P. Thoma R. J. Huang M. (2006). Impairment on the hippocampal-dependent virtual Morris water task in schizophrenia. *Schizophr. Res.* 87 67–80. 10.1016/j.schres.2006.05.021 16844347

[B30] Harvey C. D. Coen P. Tank D. W. (2012). Choice-specific sequences in parietal cortex during a virtual-navigation decision task. *Nature* 484 62–68. 10.1038/nature10918 22419153 PMC3321074

[B31] Hatton D. D. Hooper S. R. Bailey D. B. Skinner M. L. Sullivan K. M. Wheeler A. (2002). Problem behavior in boys with fragile X syndrome. *Am. J. Med. Genet.* 108 105–116. 10.1002/ajmg.10216 11857559

[B32] Hatton D. D. Sideris J. Skinner M. Mankowski J. Bailey D. B. Roberts J. (2006). Autistic behavior in children with fragile X syndrome: prevalence, stability, and the impact of FMRP. *Am. J. Med. Genet. A* 140 1804–1813. 10.1002/ajmg.a.31286 16700053

[B33] Hebb D. O. Williams K. (1946). A method of rating animal intelligence. *J. Gen. Psychol.* 34 59–65. 10.1080/00221309.1946.10544520 21015350

[B34] Heft H. (1979). The role of environmental features in route-learning: two exploratory studies of way-finding. *Environ. Psychol. Nonverbal Behav.* 3 172–185. 10.1007/BF01142591

[B35] Hock B. Bunsey M. (1998). Differential effects of dorsal and ventral hippocampal lesions. *J. Neurosci.* 18 7027–7032. 9712671 10.1523/JNEUROSCI.18-17-07027.1998 PMC6792982

[B36] Huber K. M. Gallagher S. M. Warren S. T. Bear M. F. (2002). Altered synaptic plasticity in a mouse model of fragile X mental retardation. *Proc. Natl. Acad. Sci. U.S.A.* 99 7746–7750. 10.1073/pnas.122205699 12032354 PMC124340

[B37] Hunsaker M. R. Tran G. T. Kesner R. P. (2008). A double dissociation of subcortical hippocampal efferents for encoding and consolidation/retrieval of spatial information. *Hippocampus* 18 699–709. 10.1002/hipo.20429 18493950

[B38] Hyvarinen J. Poranen A. (1974). Function of the parietal associative area 7 as revealed from cellular discharges in alert monkeys. *Brain* 97 673–692. 10.1093/brain/97.1.673 4434188

[B39] Irwin S. A. Galvez R. Greenough W. T. (2000). Dendritic spine structural anomalies in fragile-X mental retardation syndrome. *Cereb. Cortex* 10 1038–1044. 10.1093/cercor/10.10.1038 11007554

[B40] Jakala P. Hanninen T. Ryynanen M. Laakso M. Partanen K. Mannermaa A. (1997). Fragile-X: neuropsychological test performance, CGG triplet repeat lengths, and hippocampal volumes. *J. Clin. Invest.* 100 331–338. 10.1172/JCI119538 9218509 PMC508195

[B41] Jansen-Osmann P. Fuchs P. (2006). Wayfinding behavior and spatial knowledge of adults and children in a virtual environment. *Exp. Psychol.* 53 171–181. 10.1027/1618-3169.53.3.171 16955726

[B42] Karmiloff-Smith A. (1998). Development itself is the key to understanding developmental disorders. *Trends Cogn. Sci.* 2 389–398. 10.1016/S1364-6613(98)01230-3 21227254

[B43] Karmiloff-Smith A. (2009). Nativism versus neuroconstructivism: rethinking the study of developmental disorders. *Dev. Psychol.* 45 56–63. 10.1037/a0014506 19209990

[B44] Klusek J. Hunt A. W. Mirrett P. L. Hatton D. D. Hooper S. R. Roberts J. E. (2015). Reading and phonological skills in boys with fragile X syndrome. *J. Autism Dev. Disord.* 45 1699–1711. 10.1007/s10803-014-2328-y 25448919 PMC4442735

[B45] Koekkoek S. K. Yamaguchi K. Milojkovic B. A. Dortland B. R. Ruigrok T. J. Maex R. (2005). Deletion of FMR1 in Purkinje cells enhances parallel fiber LTD, enlarges spines, and attenuates cerebellar eyelid conditioning in fragile X syndrome. *Neuron* 47 339–352. 10.1016/j.neuron.2005.07.005 16055059

[B46] Kogan C. S. Boutet I. Cornish K. Graham G. E. Berry-Kravis E. Drouin A. (2009). A comparative neuropsychological test battery differentiates cognitive signatures of Fragile X and down syndrome. *J. Intellect. Disabil. Res.* 53 125–142. 10.1111/j.1365-2788.2008.01135.x 19054268

[B47] Kogan C. S. Boutet I. Cornish K. Zangenehpour S. Mullen K. T. Holden J. J. A. (2004b). Differential impact of the FMR1 gene on visual processing in fragile X syndrome. *Brain* 127 591–601. 10.1093/brain/awh069 14736752

[B48] Kogan C. S. Bertone A. Cornish K. Boutet I. Der Kaloustian V. M. Andermann E. (2004a). Integrative cortical dysfunction and pervasive motion perception deficit in fragile X syndrome. *Neurology* 63 1634–1639. 15534248 10.1212/01.wnl.0000142987.44035.3b

[B49] Kooy R. F. (2003). Of mice and the fragile X syndrome. *Trends Genet.* 19 148–154. 10.1016/S0168-9525(03)00017-9 12615009

[B50] Lightbody A. A. Hall S. S. Reiss A. L. (2006). Chronological age, but not FMRP levels, predicts neuropsychological performance in girls with fragile X syndrome. *Am. J. Med. Genet. B* 141B 468–472. 10.1002/ajmg.b.30307 16741913 PMC2663575

[B51] Lind S. E. Williams D. M. Raber J. Peel A. Bowler D. M. (2013). Spatial navigation impairments among intellectually high-functioning adults with autism spectrum disorder: exploring relations with theory of mind, episodic memory, and episodic future thinking. *J. Abnorm. Psychol.* 122 1189–1199. 10.1037/a0034819 24364620 PMC3906800

[B52] MacLeod L. S. Kogan C. S. Collin C. A. Berry-Kravis E. Messier C. Gandhi R. (2010). A comparative study of the performance of individuals with fragile X syndrome and Fmr1 knockout mice on Hebb-Williams mazes. *Genes Brain Behav.* 9 53–64. 10.1111/j.1601-183X.2009.00534.x 19796132

[B53] McDuffie A. Thurman A. J. Hagerman R. J. Abbeduto L. (2015). Symptoms of autism in males with fragile X syndrome: a comparison to nonsyndromic ASD using current ADI-R scores. *J. Autism Dev. Disord.* 45 1925–1937. 10.1007/s10803-013-2013-6 24414079 PMC4096070

[B54] McGonigle P. Ruggeri B. (2014). Animal models of human disease: challenges in enabling translation. *Biochem. Pharmacol.* 87 162–171. 10.1016/j.bcp.2013.08.006 23954708

[B55] Meunier M. Saint-Marc M. Destrade C. (1986). The Hebb-Williams test to assess recovery of learning after limbic lesions in mice. *Physiol. Behav.* 37 909–913. 10.1016/S0031-9384(86)80011-7 3786484

[B56] Miller L. J. McIntosh D. N. McGrath J. Shyu V. Lampe M. Taylor A. K. (1999). Electrodermal responses to sensory stimuli in individuals with fragile X syndrome: a preliminary report. *Am. J. Med. Genet.* 83 268–279. 10.1002/(SICI)1096-8628(19990402)83 10208160

[B57] Mineur Y. S. Sluyter F. de Wit S. Oostra B. A. Crusio W. E. (2002). Behavioral and neuroanatomical characterization of the Fmr1 knockout mouse. *Hippocampus* 12 39–46. 10.1002/hipo.10005 11918286

[B58] Mitchell S. Rawlins J. Steward O. Olton D. (1982). Medial septal area lesions disrupt theta rhythm and cholinergic staining in medial entorhinal cortex and produce impaired radial arm maze behaviour in rats. *J. Neurosci.* 2 292–302. 7062110 10.1523/JNEUROSCI.02-03-00292.1982 PMC6564337

[B59] Moffat S. D. (2009). Aging and spatial navigation: what do we know and where do we go? *Neuropsychol. Rev.* 19 478–489. 10.1007/s11065-009-9120-3 19936933

[B60] Moffat S. D. Resnick S. M. (2002). Effects of age on virtual environment place navigation and allocentric cognitive mapping. *Behav. Neurosci.* 116 851–859. 10.1037/0735-7044.116.5.851 12369805

[B61] Morris R. Garrud P. Rawlins J. O’Keefe J. (1982). Place navigation impaired in rats with hippocampal lesions. *Nature* 297 681–683. 10.1038/297681a0 7088155

[B62] Okada K. Okaichi H. (2009). Functional differentiation and cooperation among the hippocampal subregions in rats to effect spatial memory processes. *Behav. Brain Res.* 200 181–191. 10.1016/j.bbr.2009.01.011 19378463

[B64] Packard M. G. McGaugh J. L. (1996). Inactivation of hippocampus or caudate nucleus with lidocaine differentially affects expression of place and response learning. *Neurobiol. Learn. Mem.* 65 65–72. 10.1006/nlme.1996.0007 8673408

[B65] Pollard J. S. Lysons A. M. (1969). Water deprivation and performance in the closed field test. *Br. J. Psychol.* 60 225–231. 10.1111/j.2044-8295.1969.tb01195.x

[B66] Rabinovitch M. S. Rosvold H. E. (1951). A closed-field intelligence test for rats. *Can. J. Psychol.* 5 122–128. 10.1037/h0083542 14870071

[B67] Robaey P. McKenzie S. Schachar R. Boivin M. Bohbot V. D. (2016). Stop and look! Evidence for a bias towards virtual navigation response strategies in children with ADHD symptoms. *Behav. Brain Res.* 298(Pt A) 48–54. 10.1016/j.bbr.2015.08.019 26310386

[B68] Rogers J. L. Kesner R. P. (2006). Lesions of the dorsal hippocampus or parietal cortex differentially affect spatial information processing. *Behav. Neurosci.* 120 852–860. 10.1037/0735-7044.120.4.852 16893291

[B69] Schapiro M. Murphy D. Hagerman R. Azari N. Alexander G. Miezejeski C. (1995). Adult fragile X syndrome: neuropsychology, brain anatomy, and metabolism. *Am. J. Med. Genet.* 60 480–493. 10.1002/ajmg.1320600603 8825884

[B70] Shore D. I. Stanford L. MacInnes W. J. Brown R. E. Klein R. M. (2001). Of mice and men: virtual Hebb-Williams mazes permit comparison of spatial learning across species. *Cogn. Affect. Behav. Neurosci.* 1 83–89. 10.3758/CABN.1.1.83 12467105

[B71] Sjoberg E. A. (2017). Logical fallacies in animal model research. *Behav. Brain Funct.* 13:3. 10.1186/s12993-017-0121-8 28202023 PMC5312558

[B72] Smith A. D. (2015). Spatial navigation in autism spectrum disorders: a critical review. *Front. Psychol.* 6:31. 10.3389/fpsyg.2015.00031 25667579 PMC4304163

[B73] Spiers H. Maguire E. (2007). A navigational guidance system in the human brain. *Hippocampus* 17 618–626. 10.1002/hipo.20298 17492693 PMC2570439

[B74] Sullivan K. Hatton D. Hammer J. Sideris J. Hooper S. Ornstein P. (2006). ADHD symptoms in children with FXS. *Am. J. Med. Genet. A* 140A 2275–2288. 10.1002/ajmg.a.31388 17022076

[B75] Van Dam D. D’Hooge R. Hauben E. Reyniers E. Gantois I. Bakker C. E. (2000). Spatial learning, contextual fear conditioning and conditioned emotional response in Fmr1 knockout mice. *Behav. Brain Res.* 117 127–136. 10.1016/S0166-4328(00)00296-5 11099766

[B76] van der Staay J. F. Arndt S. S. Nordquist R. E. (2010). The standardization–generalization dilemma: a way out. *Genes Brain Behav.* 9 849–855. 10.1111/j.1601-183X.2010.00628.x 20662940

[B77] Verkerk A. Pieretti M. Sutcliffe J. Fu Y. Kuhl D. Pizzuti A. (1991). Identification of a gene (FMR-1) containing a CGG repeat coincident with a breakpoint cluster region exhibiting length variation in fragile X syndrome. *Cell* 65 905–914. 10.1016/0092-8674(91)90397-H 1710175

[B78] Vorhees C. V. Williams M. T. (2014). Assessing spatial learning and memory in rodents. *ILAR J.* 55 310–332. 10.1093/ilar/ilu013 25225309 PMC4240437

[B79] Waller D. Lippa Y. (2007). Landmarks as beacons and associative cues: their role in route learning. *Mem. Cogn.* 35 910–924. 10.3758/BF03193465 17910176

[B80] Watase K. Zoghbi H. Y. (2003). Modelling brain diseases in mice: the challenges of design and analysis. *Nat. Rev. Genet.* 4 296–307. 10.1038/nrg1045 12671660

[B81] Wilkniss S. M. Jones M. G. Korol D. L. Gold P. E. Manning C. A. (1997). Age-related differences in an ecologically based study of route learning. *Psychol. Aging* 12 372–375. 10.1037/0882-7974.12.2.372 9189997

[B82] Willner P. (1986). Validation criteria for animal models of human mental disorders: learned helplessness as a paradigm case. *Prog. Neuropsychopharmacol. Biol. Psychiatry* 10 677–690. 10.1016/0278-5846(86)90051-5 3809518

